# Bibliometric analysis of Italy's orthopaedics and Sports Medicine research output: Trends, impact, and comparative rankings (1996-2024)

**DOI:** 10.1016/j.jor.2026.03.003

**Published:** 2026-03-04

**Authors:** Raju Vaishya, Abhishek Vaish, Gennaro Pipino, Filippo Migliorini

**Affiliations:** a Department of Orthopaedics and Joint Replacement Surgery, Indraprastha Apollo Hospitals, New Delhi, India; b Department of Orthopaedic, Villa Erbosa Hospital, Bologna, Italy; c Department of Trauma and Reconstructive Surgery, University Hospital of Halle, Martin-Luther University Halle-Wittenberg, 06097, Halle (Saale), Germany; d Department of Life Sciences, Health, and Health Professions, Link Campus University, Via del Casale di San Pio V, 00165, Rome, Italy; e Department of Orthopaedic and Trauma Surgery, Eifelklinik St.Brigida, Kammerbruchstr. 8, 52152, Simmerath, Germany

**Keywords:** Bibliometrics, Orthopaedics, Sports medicine, Italy, Publications, Citations, H-index

## Abstract

**Background and aims:**

Orthopaedics and Sports Medicine (OSM) research plays a central role in advancing musculoskeletal healthcare. Although previous bibliometric studies have evaluated regional and continental trends, a detailed and standardised assessment of Italy's long-term contribution remains limited. This study aimed to comprehensively analyse Italy's OSM research output, impact, and publishing landscape from 1996 to 2024 using harmonised SCImago indicators.

**Methods:**

A descriptive bibliometric analysis was conducted using data retrieved from the SCImago Journal & Country Rank database, derived from Scopus-indexed sources. Publications classified under the “Orthopaedics and Sports Medicine” category (code 2732) and affiliated with Italy were identified. Annual and cumulative document counts, total citations, self-citations, H-index, and citations per publication (CPP) were extracted. Multinational publications were assigned to Italy based on author affiliation. All data were independently collected and cross-validated by two reviewers. Citation indicators were recalculated to ensure internal consistency.

**Results:**

Italy's annual OSM output increased from 356 publications in 1996 to 1711 in 2024, corresponding to a 4.8-fold growth and a cumulative total of 22,129 documents. In 2024, Italy ranked ninth globally and fourth in Western Europe, accounting for 9.55% of worldwide output. Impact indicators were substantial, with an H-index of 211, 1845 citations in 2024, and a cumulative CPP exceeding 22, based on more than 504,000 citations. Eleven Italian journals covered all SJR quartiles, led by the Journal of Orthopaedics and Traumatology (Q1, H-index 57).

**Conclusions:**

Italy demonstrates sustained growth and high international visibility in OSM research. The application of transparent and standardised bibliometric methods strengthens the reliability of these findings and supports their relevance for research policy and strategic planning in European musculoskeletal science.

## 1 Introduction

Orthopaedics and Sports Medicine (OSM) represents a cornerstone of modern healthcare, encompassing the prevention, diagnosis, and treatment of musculoskeletal disorders, traumatic injuries, and performance optimisation in athletic populations. With an ageing global demographic and rising participation in sports, the demand for high-quality OSM research has surged, driving innovations in surgical techniques, rehabilitation protocols, and regenerative therapies.^1^^,^^2^ Bibliometric analyses have become invaluable tools for mapping research landscapes, identifying influential contributors, and highlighting disparities in productivity and impact across regions.^3^^,^^4^ Such studies have previously examined OSM output from diverse geographies, including Africa,^5^ India,^6^ Iraq,^7^ Iran,^8^ Latin America,^9^ Nepal,^10^ South Africa,^11^ SAARC countries,^12^ and broader European trends.^1^^,^^13^ These investigations reveal a global shift toward increased publication output from emerging economies, yet high-income nations maintain dominance in citation and H-index metrics.^1^^,^^14^

Within Europe, which collectively ranks second globally in OSM productivity behind North America,^14^^,^^15^ individual country contributions vary markedly, influenced by funding, institutional infrastructure, and collaborative networks.^16^ Italy, with its rich history in medical research and strong emphasis on clinical translation, has emerged as a key player, yet dedicated bibliometric appraisals of its OSM research remain scarce.^14^ Prior European analyses note Italy's steady ascent but lack granular insights into temporal trends, impact indicators, and domestic publishing ecosystems.^14^ This gap is particularly notable given Italy's potential to bridge basic science and applied orthopaedics, as evidenced by its contributions to traumatology and sports-related studies.^16^, ^17^, ^18^

The present study addresses this void by providing a comprehensive bibliometric analysis of Italy's OSM research output from 1996 to 2024. Drawing on SCImago data, we evaluate publication volume, citation dynamics, H-index, and comparative rankings, while profiling leading Italian journals. By elucidating Italy's strengths and trajectories, this work aims to inform policy, foster international partnerships, and guide future research investments in OSM.^19^^,^^20^

## 2 Methods

This descriptive bibliometric study was conducted using publicly available data from the SCImago Journal & Country Rank (SJR) database (https://www.scimagojr.com/), which is derived from the Scopus database. Data were accessed on November 21, 2025, and rechecked on December 5, 2025, to ensure the stability and consistency of the retrieved indicators.

## 3 Data source and search strategy

Publications were identified within the subject category “Orthopaedics and Sports Medicine” (Scopus category code 2732), as defined by SCImago. All documents indexed under this category and affiliated with Italy between January 1, 1996 and December 31, 2024, were included. No language restrictions were applied. The country filter “Italy” was applied through the SJR country ranking interface. Documents were assigned to Italy based on at least one author's institutional affiliation with an Italian institution, in accordance with SCImago attribution criteria.

## 4 Document classification and eligibility

Only peer-reviewed journal articles indexed by Scopus were included. Editorials, conference abstracts, letters, errata, and non-indexed materials were excluded by default through the SJR filtering system. All included documents were classified as citable items according to SCImago definitions. Multinational publications were counted for Italy when at least one co-author reported an Italian affiliation. These documents were considered in accordance with the full-counting method adopted by SCImago, which assigns one full publication credit to each contributing country.

## 5 Data extraction procedures

Data extraction was independently performed by two reviewers using standardised extraction forms. The following indicators were retrieved for each year and cumulatively:

• Number of documents
• Number of citable documents
• Total citations received
• National self-citations
• H-index

All extracted data were cross-checked between reviewers. Discrepancies were resolved through joint re-extraction and consensus. When inconsistencies were detected, the original SJR database entries were reconsulted.

## 6 Definition of bibliometric indicators

Total citations (TC) were defined as the number of citations received by Italian-affiliated OSM publications within the Scopus database at the time of data retrieval. Self-citations were defined as citations originating from publications that included at least one Italian-affiliated author, in line with SCImago methodology. The H-index was defined as the maximum value h such that h publications received at least h citations. Citations per publication (CPP) were calculated as the ratio between total citations and total number of documents for the corresponding period:

CPP = Total citations/Total publications. To ensure internal consistency, CPP and self-citation rates were recalculated manually and compared with SJR-reported values.

## 7 Analysis of Italian journals

Italian journals classified under the “Orthopaedics and Sports Medicine” category were identified using the SJR journal ranking tool and filtered by country of publication. For each journal, the following variables were extracted: SJR quartile, H-index, number of published documents (2022–2024), number of citations (2022–2024), and coverage years.

## 8 Data analysis

Descriptive statistics were used to summarise publication trends, citation patterns, and growth rates. Annual and cumulative values were analysed. Relative contributions were expressed as percentages of global output. Temporal trends were visualised using line graphs. No inferential statistical analyses were performed, as the study aimed to provide an exploratory and descriptive mapping of research activity.

## 9 Ethical considerations

Ethical approval was not required, as all data were obtained from publicly accessible and anonymised sources.

## 10 Results

### 10.1 Productivity trends and growth patterns

**Fig. 1:**
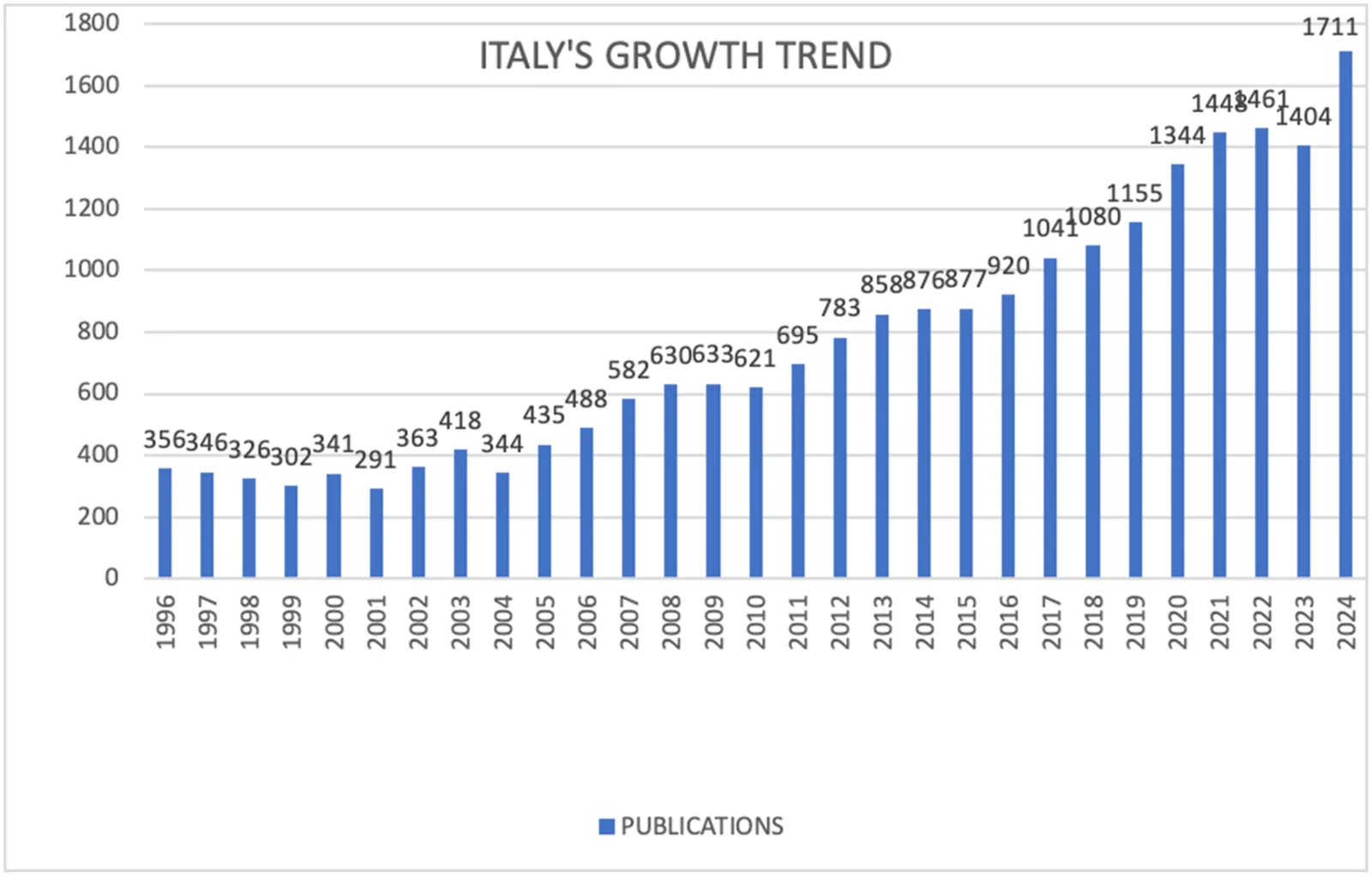
Italy's research output trend in Orthopaedic and Sports Medicine from 1996 to 2024.

Between 1996 and 2024, Italy's annual publication output in Orthopaedics and Sports Medicine increased from 356 to 1711 documents, corresponding to a cumulative growth of 381% and an average annual growth rate of approximately 5.7% (Fig. 1). Three developmental phases were identified: gradual expansion (1996–2005), consolidation (2006–2014), and accelerated growth (2015–2024), during which annual output nearly doubled from 877 to 1711 publications. During the last decade, Italy's share of global OSM output increased steadily, reaching 9.55% in 2024 (Fig. 1). Cumulatively, 22,129 publications were produced, ranking Italy ninth worldwide and fourth in Western Europe. The proportion of citable documents remained consistently above 95%.

### 10.2 Citation performance and impact indicators

In 2024, Italian OSM publications received 1845 citations, corresponding to a citations-per-publication ratio (CPP) of 1.08 (Table 1). Cumulative citations exceeded 504,000, resulting in a long-term CPP greater than 22. Self-citations accounted for 676 citations in 2024, representing approximately 20% of total citations (Table 1). This proportion remained stable over time. The national H-index reached 211, positioning Italy among the leading European countries.

**Table 1:** Comparative productivity of the top 10 european countries in orthopaedic and sports medicine (2024).

European Rank	Country	Publications	Citable documents	Citations	Self-citations	Citations per publication	H-index
**1**	United Kingdom	3127	2915	2915	774	0.93	346
**2**	Germany	2496	2286	1822	561	0.73	254
**3**	Spain	1959	1877	2077	618	1.06	193
**4**	Italy	1711	1628	1845	676	1.08	211
**5**	France	1706	1588	1169	393	0.69	247
**6**	Switzerland	1226	1137	1172	257	0.96	263
**7**	Netherlands	1075	1003	1032	216	0.96	245
**8**	Sweden	673	623	630	115	0.94	245
**9**	Portugal	594	577	537	136	0.9	116
**10**	Norway	539	500	525	109	0.97	201

### 10.3 International and regional positioning

In 2024, Italy ranked ninth globally and fourth in Western Europe in publication volume (Table 2). Italy demonstrated one of the highest CPP values among European countries and maintained a favourable balance between productivity and impact (Table 1). At the global level, Italy's CPP exceeded that of several emerging research systems and was comparable to that of Australia and Canada (Table 2).

**Table 2:** Bibliometric profile of the top 20 countries in orthopaedic and sports medicine research.

Rank	Country	Region	Publications	Citable documents	Citations	Self-citations	Citations per publication	H index
**1**	United States	Northern America	13338	12418	8322	3923	0.62	520
**2**	China	Asiatic Region	5933	5728	3035	1786	0.51	152
**3**	United Kingdom	Western Europe	3127	2915	2915	774	0.93	346
**4**	Germany	Western Europe	2496	2286	1822	561	0.73	254
**5**	Japan	Asiatic Region	2227	2163	1237	366	0.56	227
**6**	Australia	Pacific Region	2086	1951	2080	546	1	317
**7**	Spain	Western Europe	1959	1877	2077	618	1.06	193
**8**	Canada	Northern America	1916	1782	1656	368	0.86	307
***9***	*Italy*	*Western Europe*	*1711*	*1628*	*1845*	*676*	*1.08*	*211*
**10**	France	Western Europe	1706	1588	1169	393	0.69	247
**11**	Brazil	Latin America	1491	1424	842	194	0.56	152
**12**	Switzerland	Western Europe	1226	1137	1172	257	0.96	263
**13**	Turkey	Middle East	1212	1159	866	226	0.71	108
**14**	India	Asiatic Region	1167	1056	594	161	0.51	101
**15**	South Korea	Asiatic Region	1078	1039	576	169	0.53	159
**16**	Netherlands	Western Europe	1075	1003	1032	216	0.96	245
**17**	Iran	Middle East	726	700	454	100	0.63	86
**18**	Indonesia	Asiatic Region	722	722	1743	1508	2.41	34
**19**	Sweden	Western Europe	673	623	630	115	0.94	245
**20**	Russian Federation	Eastern Europe	631	625	103	61	0.16	58

### 10.4 Performance of Italian journals

Eleven Italian journals were indexed in the OSM category. Between 2022 and 2024, they collectively published over 450 documents and generated more than 3100 citations (Table 3).

**Table 3:** Leading Italian orthopaedic and sports medicine journals and performance (2024).

Journal	Publisher	SJR Quartile	H-index	Citations (3 Years)	Docs (2024)	Coverage Years
***Journal of Orthopaedics and Traumatology***	Springer-Verlag Italia	Q1	57	1699	175	2002–2025
***Hip International***	Wichtig Publishing Srl	Q2	50	673	103	1997–2025
***European Journal of Translational Myology***	Page Press Publications	Q3	19	222	62	2018–2024
***Sport Sciences for Health***	Springer-Verlag Italia	Q3	27	276	40	2004–2025
***Muscles Ligaments and Tendons Journal***	EDRA S.p.A	Q3	27	123	27	2011–2024
***Minerva Orthopedics***	Edizioni Minerva Medica	Q4	14	87	45	2021–2024
***European Journal of Musculoskeletal Diseases***	Biolife Publisher	Q4	14	87	45	2019–2024

The Journal of Orthopaedics and Traumatology showed the highest impact, with 1699 citations from 175 publications, followed by the consistent performance of HIP International and Q3 interdisciplinary journals (Table 3). The distribution across SJR quartiles remained stable, indicating a diversified national publishing ecosystem.

### 10.5 Structural indicators of research quality

The ratio between cumulative publications and H-index, together with the stability of CPP and self-citation rates, reflects sustained research quality and international integration within the global OSM research community.

## 11 Discussion

Italy's OSM research journey, as revealed by this analysis, reinforces a narrative of sustained expansion and scholarly influence, with publications surging 4.8-fold from 356 in 1996 to 1711 in 2024; a near-doubling in the past decade alone.^14^ This positions Italy as the ninth-largest global contributor cumulatively (22,129 documents) and fourth in Western Europe for 2024 output, trailing only the UK, Germany, and Spain ^14^, ^15^. Such growth outpaces many peers; for instance, while India's OSM publications have risen notably over the last decade,^6^ Italy's absolute volume and European ranking reflect deeper infrastructural investments in academic and clinical centres.^18^^,^ ^21^ Globally, Italy's 9.55% share of 2024 output lags behind the US (27.6%) and China, but signals a maturing ecosystem capable of rivalling Asian surges documented in broader trends.^1^^,^^19^

When interpreted in the context of existing national and regional bibliometric analyses, Italy's performance in OSM reflects a pattern of sustained and structurally stable growth. Studies from emerging research systems, including India, Iran, and several African countries, have reported rapid recent increases in publication volume, often associated with lower citation densities and higher self-citation rates.^5^^,^^6^^,^^8^ In contrast, Italy demonstrates a more gradual expansion, combined with consistently favourable H-index and CPP values, suggesting long-term integration into international research networks. European analyses have highlighted the dominance of the UK and Germany, alongside heterogeneous development trajectories across Southern and Eastern Europe.^14^^,^^18^ Within this framework, Italy occupies an intermediate position between high-output systems and smaller national environments, showing greater temporal continuity and citation stability than many Mediterranean counterparts. Moreover, studies from Latin America and the SAARC regions have emphasised limitations in journal visibility and international dissemination.^9^^,^^12^ Italy's diversified national journal ecosystem may partly explain its stronger long-term citation performance and international visibility.

Beyond volume, Italy's impact metrics affirm its qualitative prowess: an H-index of 211 ranks among Europe's elite, while the 2024 CPP of 1.08 exceeds regional medians, and the cumulative CPP exceeds 22; evidence of enduring relevance in clinical and translational domains.^4^^,^^13^ The 1845 citations received in 2024, with a modest 20% self-citation rate, highlight robust international engagement, contrasting higher partnership in some low- and middle-income contexts.^5^^,^^7^^,^^8^ This external validation aligns with patterns in highly cited orthopaedic works, where interdisciplinary appeal and methodological rigour drive longevity.^4^^,^^20^ Cumulatively, over 504,000 citations since 1996 not only eclipse outputs from regions such as Latin America^9^ and SAARC nations,^12^ but also position Italy as a citation hub within Europe.^14^^,^^17^

A standout feature is Italy's domestic journal landscape, comprising 11 OSM-focused outlets spanning all quartiles and supported by publishers such as Springer-Verlag Italia and Edizioni Minerva Medica.^15^^,^^22^ The Q1 *Journal of Orthopaedics and Traumatology* (H-index 57; 1700 citations from 175 recent papers) exemplifies high-impact dissemination, while Q2-Q4 venues like *HIP International* and *Minerva Orthopaedics* nurture emerging themes in rehabilitation and soft-tissue research.^15^^,^^23^ This diversity promotes inclusivity for early-career scholars and specialised topics, mirroring evolutions in journal rankings globally.^6^^,^^15^ Compared to journal-centric analyses in other locales,^9^^,^^24^ Italy's ecosystem enhances visibility and collaboration, potentially amplifying altmetric scores in an era of open-access mandates.^20^

These findings illuminate Italy's research leadership in OSM, yet challenges persist. This analysis relies exclusively on SCImago data derived from the Scopus database. Although Scopus provides broad international coverage, it does not fully capture publications indexed in regional, local, or non-English databases, potentially leading to partial underrepresentation of certain research outputs. Consequently, national productivity may be underestimated, particularly for journals with limited international visibility. Indexing and classification bias may influence bibliometric indicators. Journal inclusion criteria, subject categorisation, and periodic reclassification within Scopus may affect longitudinal comparability. Moreover, the full-counting method applied to multinational publications may overestimate national contributions in highly collaborative studies. Bibliometric indicators primarily reflect quantitative impact and citation behaviour rather than intrinsic scientific quality. Citation counts and H-index values may be influenced by self-citation practices, disciplinary citation norms, and topical popularity, limiting their capacity to assess methodological rigour, clinical relevance, or translational value. Finally, the absence of article-level qualitative assessment precludes evaluation of study design, evidence level, and clinical applicability. Future investigations integrating multiple databases and qualitative appraisal frameworks may provide a more comprehensive representation of national research performance. Future efforts should prioritise multicenter trials and South-North partnerships,^14^^,^^18^ leveraging Italy's H-index legacy to address unmet needs in traumatology and oncology.^13^^,^^16^ This bibliometric profile reinforces Italy's role as a European anchor in OSM, ensuring amplified contributions in the coming decades.^17^

## 12 Conclusion

This bibliometric analysis provides a comprehensive, methodologically transparent overview of Italy's contributions to Orthopaedics and Sports Medicine research from 1996 to 2024. The sustained 4.8-fold increase in publication output, combined with a high H-index and stable citation performance, reflects the progressive consolidation of Italy's academic and clinical research infrastructure. By applying standardised definitions, independent data extraction, and consistent recalculation of citation indicators, the present study ensures reproducibility and internal coherence of bibliometric metrics. Italy's diversified national journal ecosystem and strong international visibility further support its role as a leading contributor within the European research landscape. These findings provide a reliable benchmark for future comparative studies and may inform national and international strategies to strengthen collaborative networks, allocate research funding, and advance innovation in musculoskeletal science.

## Consent to participate

Not applicable.

## Consent to publish

Not applicable.

## Guardian/patient's consent

Not applicable.

## Ethical approval

This study complies with ethical standards.

## Registration and protocol

The present review was not registered.

## Availability of data and materials

The datasets generated during and/or analysed during the current study are available throughout the manuscript.

## Ethical statement

This study complies with ethical standards.

## Credit author statement

RV: writing; AV: writing; GP: writing; FM: writing. All authors have agreed to the final version to be published and agree to be accountable for all aspects of the work.

## Use of AI

Language editing was performed using Grammarly (Superhuman Platform Inc., San Francisco, CA, USA).

## Funding statement

The authors received no financial support for the research, authorship, and/or publication of this article.

## Conflict of interest

The authors declare that they have no competing interests in this article.
